# Hypoxia-specific targets in cancer therapy: role of splice variants

**DOI:** 10.1186/1741-7015-8-45

**Published:** 2010-07-12

**Authors:** Dirk Vordermark

**Affiliations:** 1 Department of Radiation Oncology, Martin Luther University Halle-Wittenberg, Halle, Germany

## Abstract

Tumour hypoxia is a well known adverse prognostic factor in the treatment of solid tumours. Hypoxia-inducible factor 1α (HIF-1α), a transcription factor subunit regulating a large number of hypoxia-responsive genes, is considered an attractive target for novel treatment approaches, due to a frequently reported association between HIF-1α overexpression and poor outcome in clinical series. This month in *BMC Medicine*, Dales *et al*. report on splice variants of HIF-1α in fresh frozen tissue samples of early human breast cancer, finding an association of mRNA levels of the variant HIF-1α^TAG ^with adverse clinical factors (lymph node status, hormone receptor status) and poor metastasis-free survival. This preliminary study addresses the possibility that specific targeting of individual isoforms resulting from alternative splicing may play a role in HIF-1-directed treatment approaches.

See research article: http://www.biomedcentral.com/1741-7015/8/44

## Background

This month in *BMC Medicine*, Dales and coworkers report on the expression of hypoxia-inducible factor 1α (HIF-1α) splice variants in human breast cancer [1]. This work represents an early and preliminary investigation that may become part of a process leading to further individualisation of cancer therapy, specifically addressing the role of hypoxic tumour cells.

## Discussion

Low oxygenation of tumour cells is a well known adverse prognostic factor in cancer treatment. It occurs due to (a) rapid tumour growth with resulting long diffusion distances from the nearest blood vessel ('diffusion-limited hypoxia'), as well as (b) the chaotic structure of pathological tumour vessels and resulting inadequate perfusion in part of these vessels ('perfusion-limited hypoxia') [2]. It was established mainly in the 1990 s that a low pretreatment intratumoural partial oxygen pressure (pO_2_), as determined by needle electrode measurement, is associated with a poor outcome of treatment, in particular radiotherapy but also surgical treatment, of cervical cancer or head and neck cancer [3,4]. This association has been explained by the reduced ability of ionizing radiation to produce DNA damage in the absence of oxygen as well as, more recently, by an increased potential of hypoxic tumour cells for proliferation, invasion, metastasis and angiogenesis [2].

For decades, investigators have attempted to overcome the treatment resistance of hypoxic tumours in clinical trials, for example by adding so-called 'hypoxic radiosensitiser' drugs to the regimens or introducing hyperbaric oxygen. Although many of the individual trials were negative, a modern meta-analysis confirms the efficacy of hypoxia-directed treatments [5]. While previous strategies were directed at all patients with a given tumour diagnosis, more modern approaches combine (a) the selection of patients with particularly hypoxic tumours and (b) the addition of hypoxia-specific treatment modalities to standard radiotherapy/chemotherapy only in these subgroups.

Determining tumour oxygenation by needle electrode measurements has not been fully accepted in clinical practice and less invasive methods have been proposed: These include the immunohistochemical detection of 'exogenous hypoxia markers' (2-nitroimidazole derivatives such as pimonidazole) injected intraveneously before a biopsy, the imaging of hypoxic tumour areas by nuclear medicine methods (for example, F-misonidazole positron emission tomography) or even the measurement of proposed secreted hypoxia markers (for example, osteopontin) in the patient plasma [6-8].

The transcription factor HIF-1 is a central regulator of the physiological or pathophysiological response of mammalian cells to low oxygen levels and has so far been described to regulate hundreds of genes in a hypoxia-dependent manner, many of which are growth factors or involved in cell proliferation, metabolism or vessel formation [9]. HIF-1 is a heterodimer consisting of one of the two α subunits (HIF-1α or HIF-2α) and HIF-1β. Under hypoxic conditions, the oxygen-sensitive subunit HIF-1α is not degraded via ubiquitylation but rather stabilises, translocates to the nucleus, heterodimerises with constitutively expressed HIF-1β and binds, in the presence of cofactors, to the hypoxia-responsive elements of HIF-1-regulated genes. HIF-1 is thus assumed to be regulated mostly by protein degradation.

HIF-1α protein itself and HIF-1-regulated proteins, for example, carbonic anhydrase IX (CA IX) have been studied by immunohistochemistry in paraffin-embedded tumour material as potential 'endogenous hypoxia markers'. Due to the availability of such material, a large number of retrospective analyses of series with long-term clinical outcome were published and, despite some concern about the reproducibility of HIF-1α staining, significant associations between a strong expression of such HIF-1-related proteins and poor prognosis was seen in the majority of studies on a wide range of solid tumour entities [10]. Such observations were also made in breast cancer [11,12], the topic now studied by Dales *et al*.

Despite its negative prognostic relevance, tumour hypoxia has also been discussed as an opportunity for tumour-specific treatment approaches, for low pO_2 _levels as found in solid tumours very rarely occur in normal tissues [13]. Therefore, a prognostically and mechanistically relevant gene or gene product such as HIF-1α or downstream genes may serve at the same time as an indicator of hypoxic treatment resistance (and therefore be used for patient selection) and as a therapeutic target (in a group thus selected). Inhibitors of the HIF-1 pathway have been grouped into inhibitors of transcription (for example, topoisomerase 2 inhibitors), inhibitors of translation (for example, topotecan, taxanes, epidermal growth factor receptor (EGFR)-targeting agents), inhibitors of DNA binding and transactivation (for example, chetomin) and promoters of degradation (for example, farnesyl transferase inhibitors) [14]. HIF-1 inhibitors have been shown *in vitro *and *in vivo *to reduce angiogenesis and tumour growth and enhance radiosensitivity [15-18]. Several HIF-1 inhibitors are now in early clinical trials.

The findings published by Dales *et al*. [1] are interesting for the further development of HIF-1 targeting approaches. The authors describe the presence of different HIF-1α splice variants in human breast cancer and non-malignant tissue samples. Splice variants result from alternative splicing, a process by which the exons of the RNA produced by transcription of a primary gene are reconnected in multiple ways, resulting in different mRNAs which may be translated into different protein isoforms. By performing real-time quantitative PCR of fresh frozen tissue, the authors demonstrate that the mRNA levels of a specific splice variant termed HIF-1α^TAG ^are associated with positive lymph node status, high tumour grade and negative oestrogen and progesterone status, as well as poor metastasis-free survival (on univariate analysis) in early breast cancer.

Although this study has a number of limitations (small sample size, limited information on patient and treatment characteristics, selection of two subgroups with good and poor metastasis-free survival rather than a homogenous cohort) and failed to demonstrate an independent prognostic role (on multivariate analysis) of HIF-1α splice variant expression, follow-up studies may advance our understanding of the role of alternative splicing in the identification of prognostic variables and therapeutic targets. In *in vitro *tumour models, small interfering RNAs (siRNAs) directed against wild-type vs individual splice variants of genes relevant for treatment resistance have produced specific effects on clonogenicity and radiosensitivity of human tumour cells [19].

## Conclusions

The data from Dales *et al*. suggest that if splice variants detectable in clinical tumour samples can reliably be related to clinical endpoints, targeting approaches directed specifically at these variants may play a role in the further individualisation of cancer treatment.

## Competing interests

The author declares that they have no competing interests.

## Pre-publication history

The pre-publication history for this paper can be accessed here:

http://www.biomedcentral.com/1741-7015/8/45/prepub

## References

[B1] Dales JP Beaufils N Silvy M Picard C Pauly V Pradel V Formisano-Tréziny C Bonnier P Giusiano S Charpin C Gabert J Hypoxia inducible factor 1 α (HIF-1α) splice variants: potential prognostic biomarkers in breast cancer BMC Med 2010 8 44 10.1186/1741-7015-8-44 PMC2917392 20624301

[B2] Ruan K Song G Ouyang G Role of hypoxia in the hallmarks of human cancer J Cell Biochem 2009 107 1053 1062 10.1002/jcb.22214 19479945

[B3] Nordsmark M Bentzen SM Rudat V Brizel D Lartigau E Stadler P Becker A Adam M Molls M Dunst J Terris DJ Overgaard J Prognostic value of tumor oxygenation in 397 head and neck tumors after primary radiation therapy. An international multi-center study Radiother Oncol 2005 77 18 24 10.1016/j.radonc.2005.06.038 16098619

[B4] Fyles A Milosevic M Hedley D Pintilie M Levin W Manchul L Hill RP Tumor hypoxia has independent predictor impact only in patients with node-negative cervix cancer J Clin Oncol 2002 20 680 687 10.1200/JCO.20.3.680 11821448

[B5] Overgaard J Hypoxic radiosensitization: adored and ignored J Clin Oncol 2007 25 4066 4074 10.1200/JCO.2007.12.7878 17827455

[B6] Hoogsteen IJ Lok J Marres HA Takes RP Rijken PF van der Kogel AJ Kaanders JH Hypoxia in larynx carcinomas assessed by pimonidazole binding and the value of CA-IX and vascularity as surrogate markers of hypoxia Eur J Cancer 2009 45 2904 2916 10.1016/j.ejca.2009.07.012 19699082

[B7] Rischin D Hicks RJ Fisher R Binns D Corry J Porceddu S Peters LJ Trans-Tasman Radiation Oncology Group Study 98.02. Prognostic significance of [18F]-misonidazole positron emission tomography-detected tumor hypoxia in patients with advanced head and neck cancer randomly assigned to chemoradiation with or without tirapazamine: a substudy of Trans-Tasman Radiation Oncology Group Study 98.02 J Clin Oncol 2006 24 2098 2104 10.1200/JCO.2005.05.2878 16648512

[B8] Overgaard J Eriksen JG Nordsmark M Alsner J Horsman MR Danish Head and Neck Cancer Study Group Plasma osteopontin, hypoxia, and response to the hypoxia sensitiser nimorazole in radiotherapy of head and neck cancer: results from the DAHANCA 5 randomised double-blind placebo-controlled trial Lancet Oncol 2005 6 757 764 10.1016/S1470-2045(05)70292-8 16198981

[B9] Semenza GL Hypoxia-inducible factor 1 (HIF-1) pathway Sci STKE 2007 407 cm8 10.1126/stke.4072007cm8 17925579

[B10] Bache M Kappler M Said HM Staab A Vordermark D Detection and specific targeting of hypoxic regions within solid tumors: current preclinical and clinical strategies Curr Med Chem 2008 15 322 338 10.2174/092986708783497391 18288988

[B11] Brennan DJ Jirstrom K Kronblad A Milikan RC Landberg G Duffy MJ Ryden L Gallagher WM O'Brien SL CA IX is an independent prognostic marker in premenopausal breast cancer patients with one to three positive lymph nodes and a putative marker of radiation resistance Clin Cancer Res 2006 12 6421 6431 10.1158/1078-0432.CCR-06-0480 17085655

[B12] Bos R van der Groep P Greijer AE Shvarts A Meijer S Pinedo HM Semenza GL van Diest PJ van der Wall E Levels of hypoxia-inducible factor-1a independently predict prognosis in patients with lymph node negative breast carcinoma Cancer 2003 97 1573 1581 10.1002/cncr.11246 12627523

[B13] Brown JM The hypoxic cell: a target for selective cancer therapy - eighteenth Bruce Cain Memorial Award lecture Cancer Res 1999 59 5863 5870 10606224

[B14] Koh MY Spivak-Kroizman TR Powis G Inhibiting the hypoxia response for cancer therapy: the new kid on the block Clin Cancer Res 2009 15 5945 5946 10.1158/1078-0432.CCR-09-1650 19789327 PMC2760048

[B15] Dewhirst MW Intermittent hypoxia furthers the rationale for hypoxia-inducible factor-1 targeting Cancer Res 2007 67 854 855 10.1158/0008-5472.CAN-06-4744 17283112

[B16] Kung AL Zabludoff SD France DS Friedmann SJ Tanner EA Vieira A Cornell-Kennon S Lee J Wang B Wang J Memmert K Naegeli HU Petersen F Eck MJ Bair KW Wood AW Livingston DM Small molecule blockade of transcriptional coactivation of the hypoxia-inducible factor pathway Cancer Cell 2004 6 33 43 10.1016/j.ccr.2004.06.009 15261140

[B17] Williams KJ Telfer BA Xenaki D Sheridan MR Desbaillets I Peters HJ Honess D Harris AL Dachs GU van der Kogel A Stratford IJ Enhanced response to radiotherapy in tumours deficient in the function of hypoxia-inducible factor-1 Radiother Oncol 2005 75 89 98 10.1016/j.radonc.2005.01.009 15878106

[B18] Staab A Loeffler J Said HM Diehlmann D Katzer A Beyer M Fleischer M Schwab F Baier K Einsele H Flentje M Vordermark D Effects of HIF-1 inhibition by chetomin on hypoxia-related transcription and radiosensitivity in HT 1080 human fibrosarcoma cells BMC Cancer 2007 7 213 10.1186/1471-2407-7-213 17999771 PMC2200672

[B19] Kappler M Rot S Taubert H Greither T Bartel F Dellas K Hänsgen G Trott KR Bache M The effects of knockdown of wild-type survivin, survivin-2B or survivin-delta3 on the radiosensitization in soft-tissue sarcoma cells in vitro under different oxygen conditions Cancer Gene Ther 2007 14 994 1001 10.1038/sj.cgt.7701090 17885676

